# Alternative Processing Options for Improving the Proteins Functionality by Maillard Conjugation

**DOI:** 10.3390/foods12193588

**Published:** 2023-09-27

**Authors:** Loredana Dumitrașcu, Daniela Borda, Iuliana Aprodu

**Affiliations:** Faculty of Food Science and Engineering, Dunarea de Jos University of Galati, 111 Domneasca Str., 800008 Galati, Romania; loredana.dumitrascu@ugal.ro (L.D.); daniela.borda@ugal.ro (D.B.)

**Keywords:** proteins, Maillard reactions, alternative processing techniques

## Abstract

Conjugation of the proteins with carbohydrates, occurring in the early stages of the Maillard reactions, received increased attention because of the high potential to ensure the improvement of the biological activity and functional properties of the proteins of different origins. The Maillard conjugates are conventionally formed through wet or dry heating, but the use of alternative technologies involving ultrasound, microwave, pulsed electric fields, high-pressure, or electrodynamic treatments appears to be efficient in accelerating the reaction steps and limiting the formation of toxic compounds. An overview of the mechanisms of these processing technologies, the main parameters influencing the Maillard conjugate formation, as well as their advantages and disadvantages, is provided in this paper. Different strategies employing these alternative technologies are reported in the literature: as pretreatment of the proteins, either alone or in admixture with the carbohydrates, followed by conventional heating, as a single alternative treatment step, or as a combination of heating and alternative processing. The desired functional properties of the proteins can be achieved by selecting the appropriate processing strategy and optimizing the reaction parameters. Moreover, alternative technologies can be exploited to obtain Maillard conjugates with remarkable biological activity in terms of antioxidant, antimicrobial, antihypertensive, anti-inflammatory, antimutagenic, or bifidogenic properties.

## 1. Introduction

Considered one of biology’s most versatile polymers present in foods, proteins are playing roles as nutrients and structural building blocks for stabilizing texture and maintaining the food’s qualitative attributes [1,2,3]. Proteins have a strong contribution to the development of foods with functional properties influencing the bioavailability of nutrients, equilibrium with minerals, and other bioactive compounds [2]. The functionality of the proteins is associated with their ability to form and/or stabilize gels, films, foams, emulsions, etc. while maximizing their impact on nutrition and health [3]. On the other hand, the most important drawbacks of the food proteins are attributed to their sensibility to high temperatures, pH changes, high ionic strength, and specific proteolytic and organic agents that limit their use for industrial applications [4,5]. The Maillard reaction represents one of the most exploited strategies for improving the functionality of proteins. This approach involves a complex set of interrelated non-enzymatic reactions, where the reducing sugars react with the free amino groups of proteins or peptides to form Maillard reaction products. From a chemical point of view, the Maillard reaction occurs in three stages: early, intermediate, and advanced [5]. The early stage starts with the amino–sugar condensation (reversible Schiff base) accompanied by the Amadori rearrangement. The products that resulted in this stage are colorless, do not have the capacity to absorb ultraviolet light at 280 nm, and exhibit functional properties in terms of emulsification, foaming, heat stability, and gelation. The intermediate stage is characterized by sugar dehydration, fragmentation, and amino acid degradation, whereas the advanced stage includes aldol and aldehyde–amine condensation and the formation of heterocyclic nitrogen compounds [6]. In the intermediate/advanced stages, bioactive products are formed with antioxidant and/or antimicrobial activity, heterocyclic compounds, reductones, and melanoidins, accompanied by color shifting from colorless to yellow, which is more intense in the final stage of the Maillard reaction [6,7,8]. On the other hand, toxic compounds (with mutagenic, carcinogenic, and cytotoxic activity) can also be formed in the advanced stages, but the accumulation of these compounds can be prevented by controlling the reaction conditions [5]. Detailed chemical mechanisms of the Maillard reaction are provided by recent studies reported by Naik et al. [9], Zha et al. [8], and de Oliveira et al. [5]. The physicochemical characteristics in terms of molecular size of the reactants, the molar ratio between proteins/peptides, and carbohydrates, the number of available free amino and carbonyl groups, pH, water activity, temperature, and exposure time are playing an essential role in determining the rate and extent of Maillard conjugation, with positive or negative consequences on the functionality of the resulted conjugates.

**Figure 1:**
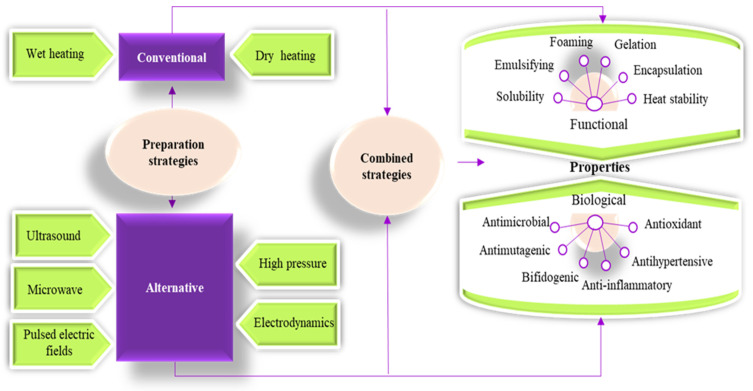
Preparation strategies for obtaining Maillard reaction compounds using alternative technologies and their impact on the functional and biological properties of the protein conjugates.

The Maillard reaction compounds can be prepared by using conventional, alternative strategies, or a combination of both, as presented in Figure 1. To the best of our knowledge, there are no review papers focusing exclusively on the use of alternative treatments for obtaining Maillard conjugates. In a world where consumer requirements for food with health-related benefits that are at the same time fresh, tasty, and natural are increasing, food scientists and engineers have seen in the use of alternative technologies a good opportunity for satisfying the new consumer demands in a sustainable manner. Therefore, this review focuses on the recent works performed to improve the functionality and bioactivity of proteins through the Maillard reaction by using alternative technologies. The review critically assesses the contribution of the main alternative technologies used for the preparation of Maillard conjugates and their effects on changing the functional and biological properties of the proteins.

## 2. Strategies to Prepare Maillard Conjugates

As shown in Figure 1, the Maillard conjugates can be prepared by involving conventional and alternative strategies. Conventional strategies are based on using heat treatment applied to the wet samples (wet heating) or dry samples (dry heating). Thus, the main difference between dry and wet heating is related to the initial dissolution of reactants (both proteins and carbohydrates) in an aqueous solution [10]. Dry heating involves heating the sample under controlled conditions of relative humidity between 60 and 80% at a temperature ranging from 60 to 130 °C for a time interval ranging from minutes to days. Wet heating has the advantage of involving lower temperatures (frequently 60–95 °C) and shorter exposure times (minutes to hours) and does not require a drying step, usually complemented by a drying technique such as freeze drying or spray drying. On the other hand, in wet heating, random thermal agitation of the water molecules promotes a more intense contact between proteins and carbohydrates compared to dry heating, which promotes protein denaturation and subsequent polymerization at high temperatures, causing a lower grafting degree. However, macromolecular crowding is a promising method for reducing protein denaturation and polymerization as it has the potential to accelerate the Maillard reactions while stabilizing protein structure [10,11]. The preparation of Maillard conjugates by conventional strategies using different sources of proteins and carbohydrates is given elsewhere [5,9,10,12,13].

**Figure 2:**
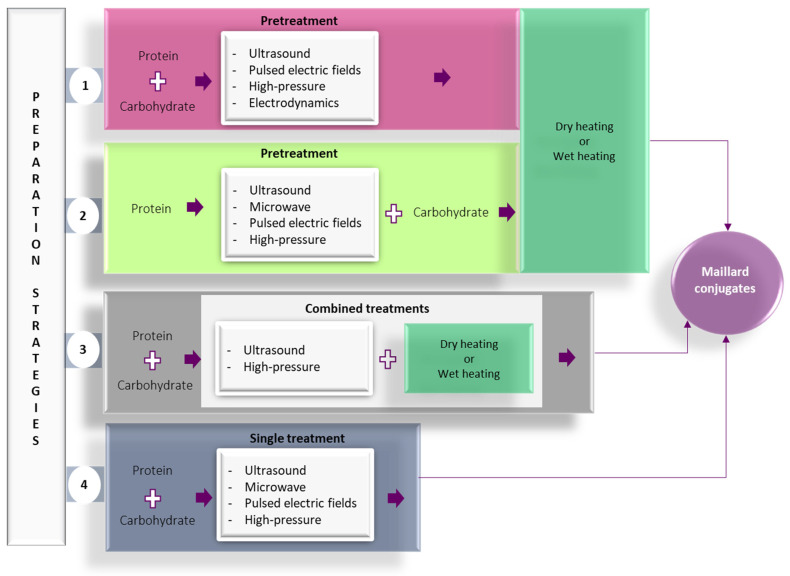
Strategies for preparation of Maillard conjugates based on the use of alternative technologies: (**1**) pretreatment of protein-carbohydrate mixture using alternative technology, followed by conventional heating; (**2**) pretreatment of the protein using alternative technologies, followed by the addition of carbohydrate and treatment with conventional heating; (**3**) the concomitant exposure of protein-carbohydrate system to combined treatment, including alternative technologies and conventional heating; (**4**) applying the alternative technology as a single treatment to the protein—carbohydrate mixtures.

**Figure 3:**
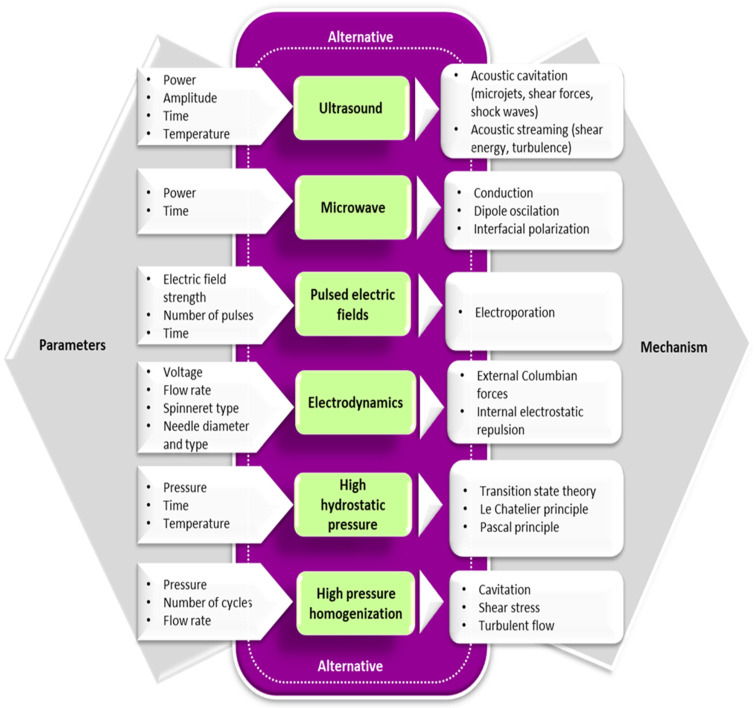
The main parameters of alternative treatments and associated mechanisms that influence the formation of the Maillard reaction products.

**Figure 4:**
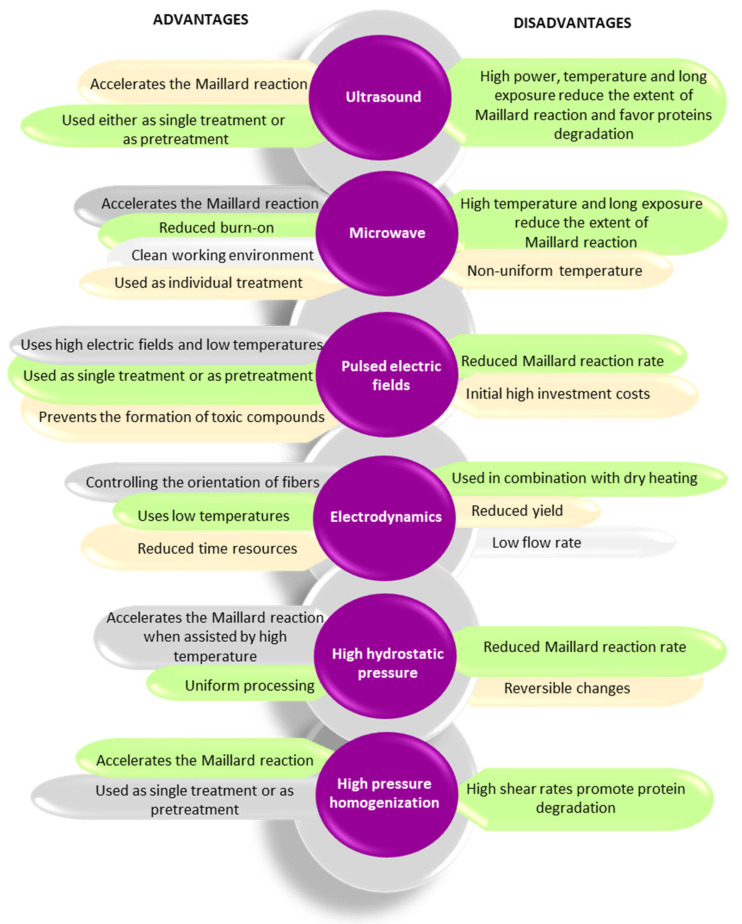
Advantages and disadvantages of using alternative technologies for producing Maillard reaction products.

In recent years, alternative technologies have been considered to have remarkable potential for complementing, or in some cases, for replacing, the conventional methods for obtaining Maillard conjugates. These technologies have the advantage of improving the functional and biological properties of protein conjugates while limiting the formation of toxic compounds like acrylamide. Often, a combination of conventional and alternative treatments is considered more cost-effective for obtaining Maillard reaction products with a positive impact on the functionality and quality of the proteins. In principle, the effects of using combined treatments on the functionality of Maillard conjugates could be additive, synergistic, or antagonistic. The schematic representation of the different strategies involving the use of alternative technologies applied for the preparation of Maillard conjugates is presented in Figure 2. Four main strategies have been used so far for obtaining Maillard conjugates employing alternative technologies. The first strategy involves the pretreatment of the protein–carbohydrate mixture using an alternative technology, followed by the application of dry or wet heating. The second strategy uses an alternative treatment to pretreat; in the initial stage, only the protein. After the pretreatment, the protein is mixed with the carbohydrate and exposed to conventional heating. This treatment is preferred when working with protein hydrolysates. The third strategy prepares the Maillard conjugates by combining the alternative with conventional heating (e.g., ultrasound (US) treatment performed at an increased temperature), while the fourth strategy prepares the conjugates by using only the alternative treatments (e.g., US treatment performed at room temperature). The selection of one of these strategies is dependent on the desired protein functionality. The main parameters of alternative treatments and the associated mechanism under which the Maillard reaction products are formed are depicted in Figure 3, whereas the advantages and disadvantages of using the alternative technologies for producing Maillard conjugates are shown in Figure 4.

### 2.1. Ultrasound (US)

US is a green, non-thermal technology that uses ultrasonic waves (above 16 kHz) to promote Maillard conjugation. During US exposure, acoustic cavitation induces local high temperatures and pressure, leading to mechanical and chemical effects that promote the formation of Maillard compounds. Acoustic cavitation is the result of the creation, expansion, and implosion of microbubbles in the medium that speed up the molecular motions of proteins and accelerate the rearrangement and expansion of protein molecules. These actions have an impact on the secondary and tertiary structure changes of proteins, which favor the exposure of reactive free amino groups of proteins, increasing the grafting speed [7]. In order to produce Maillard compounds with improved functionality, sonication has to be performed under controlled conditions. The formation of Maillard compounds during exposure to US is dependent on several factors, including power, temperature, time, and sample characteristics. Liu et al. [14] found that the US intensity between 0–150 W/cm^2^ increased the glycation rate for obtaining conjugates between α—lactalbumin and glucose, while Liu et al. [15] showed that exposure to US at 100–550 W for 25 min had no effect on the extent of glycation of β-lactoglobulin with ribose. On the other hand, too much power can decrease the glycation process due to the aggregation of proteins induced by acoustic cavitation [16]. Ma et al. [17] investigated the effects of US power (100–600 W), sonication time (15–35 min), and gum arabic concentration (0.25–1.5%) at a constant temperature of 80 °C, attempting to optimize the formation and properties of conjugates prepared with zein. The grafting degree of zein–gum arabic conjugates increased with increasing US power, exposure time, and polysaccharide concentration, reaching a maximum (32%) after 25 min of reaction at 400 W at a ratio of 2.66:1 (*w*/*w*). The increase was attributed to zein unfolding, which exposed more amino groups to participate in the Maillard reaction, and the generation of hydroxyl radicals, which enhanced the reaction between protein and polysaccharide. A further increase in power and time decreased the grafting degree due to the reduced exposure of the amino groups caused by protein aggregation (as a result of the mechanical, cavitation, and thermal effects induced by sonication on protein structure). Compared to conventional wet heating (80 °C, 20 min), the US treatment at 400 W produced conjugates with a higher degree of grafting. Similar results were reported by Jiang et al. [18] for pea protein isolate–inulin conjugates.

Zhao et al. [19] highlighted that the effect of sonication was dependent on the type of carbohydrate used in conjugation. The authors used glucose or maltose for the conjugation of soy proteins, and the effect of sonication on the glycation rate was more pronounced when using glucose. The results were attributed, on one hand, to increased reactivity of monosaccharides than disaccharides and to acoustic cavitation induced during US exposure that led to the unfolding of protein chains that exposed more reactive groups for reactions with carbonyl groups of the carbohydrate.

The ratio between reactants has to be properly chosen, as it can reach a level of saturation above which any further increase of reactants may have a negative impact on glycation, as reported by Dev et al. [20]. The authors investigated the influence of US on the conjugation of the whey protein isolate-gellan gum mixture by varying the ratio between whey protein isolate:gellan gum from 1:1 to 3:1 (*w*/*w*) and from 1:1 to 3:1 (*w*/*w*) for the gellan gum:whey protein isolate ratio, respectively. The grafting degree increased with the increase in gellan gum concentration up to a 1:2 (*w*/*w*) whey proteins:gellan gum ratio, while beyond this ratio, the degree of glycation drastically decreased. US treatment (300 W, duty cycle 50%, 60 min) was able to produce whey protein isolate–gellan gum conjugates with an increased grafting degree (17.22%) in a shorter time than the conventional method (8.41%) performed at 90 °C for 4 h. The results were associated with the US ability to depolymerize the gellan gum into smaller chains, promoting and increasing interactions that favor the extent of glycation.

The initial pH of the biopolymer system has a strong contribution to the glycation reaction; alkaline conditions promote higher intramolecular electrostatic repulsions leading to the unfolding of the proteins; and there is an increase in the number of amino groups available for the Maillard reaction. Similar to other parameters, excessive alkaline conditions have a negative impact on the grafting reaction. The influence of pH (7.0–11.0) on the formation of casein–dextran conjugates obtained using sonication as pretreatment (duty cycle 40%, 15 min) was recently tested [21]. During US exposure, the grafting degree increased up to pH 9.0 due to the formation of Schiff bases in the initial stage of the Maillard reaction, which led to the exposure of more active amino groups. Under more alkaline conditions, the condensation step of the Maillard reaction was promoted, causing a decrease in the grafting degree.

The glycation extent varies with temperature; the optimum temperature depends on the protein structure and conformation. Usually, the applied temperature has to favor protein unfolding, which increases the number of amino groups at the protein surface, making them available to react with the carbonyl groups of the carbohydrate counterpart. On the other hand, the beneficial impact of the US in the reaction system is reduced at higher temperatures, with several papers reporting a negative effect on the glycation rate. For example, in the whey proteins–gellan gum conjugates exposed to US, the grafting degree increased with the temperature up to 70 °C when the maximum exposure of the amino groups at the surface was obtained, which allowed them to react with the carbonyl ends of the gellan gum counterpart. Above 70 °C, it was reported that a smaller US power introduced in the reaction system limited the interaction between reactants, which, at the end, led to a decrease in glycation degree [20]. The optimum temperature of glycation for whey proteins–gum acacia conjugates ranged between 60 and 90 °C, for whey proteins-gum acacia conjugates between 50 and 70 °C, and from 60 °C to 80 °C for peanut protein-glucomannan conjugates [16,22,23]. On the other hand, Stanic–Vucinic et al. [24] applied US to obtain β-lactoglobulin–ribose conjugates with a high grafting degree (about 38%) under non-denaturing thermal conditions (10–15 °C).

When used as a pretreatment, US showed a positive effect on conjugation. For example, Zhao et al. [25] prepared Maillard conjugates by heating aqueous dispersions of soy protein isolate and maltodextrin after sonication at 138 W/cm^2^ at 20 kHz for up to 25 min. The sonication promoted the glycation reaction as a result of the increase in the β-type ordered secondary structure, leading to a less compact tertiary conformation of conjugates.

The exposure to US of proteins/peptide hydrolysates followed by conventional conjugation was indicated as having good potential for obtaining Maillard conjugates with improved functional and biological properties. Abdelhedi et al. [26] tested the effect of US (160 W, 30 min, 40 °C) on the evolution of glycation induced between peptides with molecular weights lower than 1 kDa obtained from enzymatic hydrolysis (with Esperase) of hound viscera proteins and sucrose. The grafting degree of the conjugates increased significantly during sonication compared to the conjugates obtained by conventional wet heating (90 °C, 2 h), due to the US’s ability to generate free radical-mediated oxidation reactions of free amino acids. Other studies reported an opposite effect of US on the glycation of whey peptides with galactose, as reported by Liu et al. [27]. The sonication of the whey peptide–galactose mixture had a negative impact on the glycation extent that decreased with increasing US exposure from 15 to 60 min, with the highest grafting degree of 21.80% being reported for the conjugates obtained by wet heating (90 °C, 2 h). The US exposure combined with wet heating promoted the exposure of free amino groups in the mixture that were previously hidden.

The US pretreatment of protein from grass carp prior to hydrolysis and conjugation with glucose proved to be very effective in promoting Maillard glycation [28]. US pretreatment at 100 W, 20 min, and 30 °C provided enough energy to change the protein secondary structure and favored the production of peptides/amino acids that accelerated the Maillard reaction. In another study, it was indicated that the formation of Maillard conjugates between the brewer’s yeast-peanut meal hydrolysate and glucose was affected by the US power, and conjugates with improved functional and sensorial properties were obtained when the US pretreatment was performed at 200 W [29]. Above this limit, the formation of Maillard products decreased and flavor characteristics were inferior because sonication inhibited the polymerization of intermediates and consequently the conversion to melanoidins. A recent study investigated the potential of using US at higher temperatures to obtain Maillard conjugates from protein hydrolysates [30]. The authors investigated the changes in the Maillard reaction between mussel meat hydrolysate and glucose/xylose under ultrasonic treatment (300 W, 2 h, pulsation mode, 60 °C), wet heating (115 °C, 2 h), and ultrasonic-assisted wet heating. The grafting degree of the conjugates prepared with ultrasonic treatment was similar to that of wet heating. The ultrasonic (300 W, 30 min, pulsation mode, 60 °C)-assisted wet heating (115 °C, 2 h) was found to have the highest Maillard reaction rate, as the acoustic cavitation and acoustic streaming induced by US exposure lead to the exposure of amino groups to the surface and the disintegration of the polypeptides into low molecular weight peptides.

Water content was shown to have a strong contribution to the glycation process, with too high or too low water content having a negative influence on the grafting degree of the Maillard reaction. Recently, the potential of US-assisted wet heating in a natural deep eutectic solvent (NADES) system for obtaining protein hydrolysate–xylose conjugates with superior functionality was investigated [31]. These eutectic solvents have the potential to regulate the glycation process by controlling the water content of biological systems exposed to Maillard glycation. The authors optimized the glycation reaction by varying the water content (0–50%), temperature (60–85 °C), US power (240–480 W), and reaction time (20–40 min). Optimized conjugates with superior functional properties were obtained when performing the Maillard reaction at 300 W, 80 °C, for 35 min, using a water content of 10%.

### 2.2. Microwave (MW)

Microwaves use electromagnetic waves in the frequency range of 300–300 GHz that induce molecular vibrations at the molecular level and changes in water polarity orientation [32]. Thus, during MW irradiation, the electromagnetic energy is transferred and converted to heat as a result of interaction with material molecules in a very short period of time [33]. The MW treatment was employed as a single treatment for obtaining Maillard conjugates from different protein sources (Table 1). Hu et al. [33] tested the effect of MW (480 W and 640 W) between 5 and 15 min on the glycation extent of a solid-state ovoalbumin–glucose mixture. The degree of glycation increased with MW power and exposure time and accelerated the interactions between the free amino groups of ovalbumin and the carbonyl groups of glucose. After exposure for 15 min at 480 W and 640 W, the temperature in the system was 93.4 °C and 109 °C, respectively. When compared to dry-heating glycation, the authors reported that MW is more efficient at improving the functionality of the proteins.

The effect of the ratio between reactants and the exposure time (15–120 min) at a MW power of 450 W on the formation of bovine serum albumin-maltodextrin conjugates was investigated by Nasrollahzadeh et al. [34]. The authors varied the weight ratio between bovine serum albumin:maltodextrin from 1:1 to 1:5 and showed that the proportion of maltodextrin had a strong effect on the Maillard reaction progression. The grafting degree reached a maximum of 40% at a 1:5 bovine serum albumin:maltodextrin weight ratio. Also, the degree of glycation increased significantly with increasing time and reached a maximum after 120 min of MW treatment. The authors concluded that MW was able to speed up the formation of Maillard reaction products compared to conventional wet heating. The MW heating was found to promote the glycation of casein with glucose and β-cyclodextrin and improve the gel properties [35]. Guan et al. [36] indicated that MW heating promoted the Maillard reaction of soy proteins–glucose conjugates and reduced the occurrence of caramelization. Moreover, the authors indicated that, compared to wet heating, exposure to MW had a lower effect on protein agglomeration, suggesting better availability of free amino groups to react with carbonyl groups. It seems that during MW exposure, the protein presents a triple state where molecules have electrons with different mutual spin orientations, with consequences on the biochemical reaction rate of the proteins [34].

### 2.3. Pulsed Electric Fields (PEF)

PEF is a nonthermal technology based on the use of short pulses of high electric fields for a very short period of time, causing an increase in internal energy in the carbon backbone of biopolymers. This energy is strong enough to weaken the hydrogen bonds between hydroxyl groups, leading to the depolymerization and decomposition of biopolymer chains and changes in the protein surface hydrophobicity due to the polarization of amino acids. Under controlled conditions, the covalent or electrostatic interactions between the proteins and carbohydrates are promoted, allowing the formation of Maillard reaction products [9,10].

Previous studies showed that PEF is able to unfold some proteins, like ovalbumin and bovine serum albumin, promoting the formation of Maillard products upon glycation [37]. PEF pretreatment (25 kV/cm, 60 μs) combined with dry glycation (55 °C, 79% relative humidity, 4 h) increased the glycation degree and promoted the reduction of IgG and IgE binding ability of the β-lactoglobulin–mannose conjugate through covalent binding with mannose [37].

Other authors have demonstrated the potential of using PEF as a single treatment for obtaining Maillard conjugates. For example, the influence of PEF (15 kV/cm, 30 kV/cm, 7.35 ms) on the glycation of the whey protein–dextran system was investigated by Sun et al. [38]. The formation of Maillard reaction products increased with increasing electric field intensity, with positive effects on the functionality of whey protein–dextran conjugates; however, the reaction rate was lower compared with conventional heating. Similar results were reported by Guan et al. [39] for the bovine serum albumin–dextran system, where the Maillard reaction was highly promoted at field intensities above 10 kV/cm. The authors explained that exposure to PEF increases proteolytic activity by forming a dynamic balance between the proteolytic and non-proteolytic forms of bovine serum albumin. Moreover, with the addition of dextran to the system, the balance was broken, increasing the number of exposed amino groups that could participate in the formation of Maillard compounds. Similar results have been reported for bovine serum albumin glycated with glucose or mannose [40]. PEF treatment performed at 10 kV/cm and 20 kV/cm had a strong effect on the conjugation rate, producing an increase from 25.92% to 30.11% in the grafting degree of the conjugates obtained with mannose. When compared to glucose, the grafting degree of conjugates prepared with mannose was always higher; however, the action mechanism was not yet elucidated.

Other reports indicated that above a threshold limit of electric field strength, the glycation degree decreases, as investigated recently by Taha et al. [41] for bovine serum albumin–starch conjugates. PEF treatment performed between 3.5 and 5.7 kV/cm promoted the Maillard reaction with a maximum degree of glycation of 8.92 ± 1.59%, while at a higher electric field strength of 8.1 kV/cm, the glycation degree decreased to 4.33% due to the lower availability of free amino groups caused by protein aggregation. Some studies mentioned the potential of PEF for obtaining Maillard conjugates with desired functionality while reducing the content of advanced glycation end products. This effect is correlated with the reduced temperature induced during PEF treatment, which lowers the extent of the glycation reaction [42].

**Table 1:** The influence of ultrasound (US), microwaves (MW), pulsed electric fields (PEF), electrospinning (ES), low pressure homogenization (LPH), dynamic high pressure microfluidization (DHPM), and high hydrostatic pressure (HHP) on the functional and biological properties of the Maillard conjugates.

Treatment Type	Protein/ Peptide Type	Carbohydrate	Ratio (*w*/*w*)	Preparation Technique According to Figure 2	Conditions	Property						Reference
						Solubility	Emulsifying	Foaming	Heat Stability	Gelation	Antioxidant Activity	
US	PPI	MD	1:1	3	US - Power: 250 W - Amplitude: 95% - Temperature: 70 °C - Time: 10–100 min - Pulsed mode: 2 s on, 2 s off	+	+					[43]
US	PPI	Dextran	1:1	3	US - Power: 200 W - Amplitude: 95% - Temperature: 80 °C - Time: 40 min - Pulsed mode: 2 s on, 2 s off	+	+					[44]
US	PEPI	Inulin	4:1 to 1:1.25	3	US - Power: 100–600 W - Temperature: 80 °C - Time: 15–35 min - Pulsed mode: 2 s on, 2 s off	+	+	+	+		+	[18]
US	WPI	GAA	4:1 to 1:1	3	US - Power: 100–700 W - Temperature: 50–90 °C - Time: 15–35 min - Pulsed mode: 2 s on, 2 s off		+		+			[16]
US	WPI	GG	1:2	3	US - Power: 100–500 W - Duty cycle: 50% - Temperature: 50–90 °C - Time: 20–80 min	+	+	+	+		+	[20]
US	SPI	MD	2:1	1	US - Power: 200 W - Time: 0–25 min - Pulsed mode: 3 s on, 1 s off Wet heating - Temperature: 95 °C - Time: 30 min					+		[25]
US	SPI	GAA	1:1	3	US - Power: 100–400 W - Temperature: 40–90 °C - Time: 5–60 min	+	+					[22]
US	SPI	C3Gal	200:1	4	US - Intensity: 106 W/cm^2^ - Time: 20 min	+			+			[45]
US	β-LG	Ribose	1:1000	4	US - Intensity: 135 W/cm^2^ - Temperature: 10–15 °C - Time: 60 min						+	[24]
US	β-CG	MD	1:1 (*v*/*v*)	3	US - Power: 450 W - Temperature: 90 °C - Time: 5–60 min		+					[46]
US	BPI	Dextran	1:1	3	US - Intensity: 545 W/cm^2^ - Temperature: 70 °C - Time: 80 min	+			+			[47]
US	MBPI	Glucose	1:1	3	US - Power: 150–450 W - Temperature: 80 °C - Time: 20 min - Pulsed mode: 2 s on, 4 s off	+	+					[48]
US	Zein	GA	2.66:1	3	US - Power: 100–600 W - Temperature: 80 °C - Time: 15–35 min	+	+		+		+	[17]
US	HVPH	Sucrose	1:1	1	US - Power: 160 W - Temperature: 40 °C - Time: 30 min Wet heating - Temperature: 90 °C - Time: 2 h						+	[26]
US	HNPH	Xylose	1:19	3	US - Power: 240–480 W - Temperature: 60–85 °C - Time: 20–40 min		+	+			+	[31]
US	MMP	Glucose/xylose	20:1	1, 3	US - Power: 300 W - Pulsed mode: 5 s on, 5 s off - Temperature: 60 °C - Time: 2 h Wet heating - Temperature: 115 °C - Time: 2 h						+	[30]
US	GCP	Glucose	50:1 (mL/g)	2	US - Power: 100 W - Temperature: 30 °C - Time: 20 min Wet heating - Temperature: 120 °C - Time: 1 h						+	[28]
US	BSGP	Maltose	2:3	2	US - Power: 500 W - Temperature: 10 °C - Time: 40 min - Pulsed mode: 4 s on, 4 s off Wet heating - Temperature: 60 °C - Time: 90 min	-		+				[49]
US	RPI	Dextran	1:1	3	- Frequency: 20–50 kHz - Temperature: 70–90 °C - Time: 0–60 min	+	+		+			[50]
US	BSA	Dextran	1:2.5	2	US - Power: 300 W - Amplitude: 0–80% - Time: 0–15 min Wet heating - Temperature: 90 °C - Time: 150 h	+	-	-				[21]
US	Casein	Dextran	1:2.5	2	US - Power: 300 W - Amplitude: 0–80% - Time: 0–15 min Wet heating - Temperature: 90 °C - Time: 150 h	+	+	+				[21]
MW	EWP	MD	20:1	4	- Power (time): 250–440 W (3–12 min); 600–900 W (2–8 min)	+/-		+				[51]
MW	OVA	Glucose	1:1	4	- Power: 480 W, 640 W - Time: 5–15 min		+				+	[33]
MW	RDP	Sodium alginate	1:1.88	4	- Power: 186 W - Temperature: 77.7 °C	+	+					[52]
MW	BSA	MD	1:1–1:5	4	- Power: 450 W, 800 W - Time: 15–120 min	+	+	+	+		+	[34]
MW	RP	Dextran	1:3	4	- Power: 100–300 W - Temperature: 70–95 °C - Time: 1–10 min	+/-	+					[53]
MW	Casein	Glucose β-cyclodextrin	1:2 1:3	4	- Power: 500 W - Time: 1–60 min					+		[35]
PEF	Glycine	Glucose	1:1	4	- Electric field strength: 0–40 kV/cm - Temperature: 39 °C - Time: 7.35 ms						+	[54]
PEF	WPI	Dextran	1:1	4	- Electric field strength: 15 kV/cm, 30 kV/cm - Temperature: 30 °C - Total time: 7.35 ms	+	+					[38]
PEF	BSA	Glucose Mannose	1:1	4	- Electric field strength: 10–20 kV/cm - Number of pulses: 73.5 - Pulse duration: 20 μs		+	+				[40]
PEF	BSA	Starch	1:1	4	- Electric field strength: 3.5–8.1 kV/cm - Temperature: 23.5–25.8 °C - Number of pulses: 10 - Pulse duration: 50 μs	+/-	+		+			[41]
ES	PEPI	MD	1:13	1	Electrospinning - Voltage: 63.8 kV - Relative humidity: 15–20% - Spinneret velocity: 55 rpm - Grounded colector velocity: 20 rpm Dry heating - Temperature: 70 °C - Relative humidity: 75% - Time: 6–24 h		+					[55]
ES	Gelatin	Glucose Sucrose Fructose	10:1	1	Electrospinning - Voltage: 15 kV - Relative humidity: 65% - Temperature: 25 °C Dry heating - Temperature: 100 °C - Relative humidity: 65% - Time: 4 h						+	[56]
LPH	PEPI	XOS	1:3	2	LPH - Pressure 80 MPa - Number of passes: 3 Wet heating - Temperature: 70 °C - Time: 20 min	+	+	+	+		+	[57]
LPH+US	PEPI	XOS	1:3	3	LPH - Pressure: 80 MPa - Number of passes: 3 US - Power: 400 W - Temperature: 70 °C - Time: 20 min	+	+	+	+		+	[57]
DHPM	LSPI	Dextran	1:2	4	- Pressure: 40–160 MPa - Number of passes: 3	+	+/-					[58]
HHP	SPI	Flaxseed gum	1:1	3	- Pressure: 0.1–300 MPa - Temperature: 60 °C - Time: 3 days	+						[59]

PPI—peanut protein isolate; MD—maltodextrin; PEPI—pea protein isolate; WPI—whey protein isolate; GA—gum arabic; GG—gellan gum; SPI—soy protein isolate; GAA—gum acacia; C3Gal—cyanidin-3-galactoside; β-LG—β-lactoglobulin; β-CG—β-conglycinin; BPI—buckwheat protein isolate; MBPI—mung bean protein isolate; HVPH—hound viscera protein hydrolysate; HNPH—*Harpadon nehereus* protein hydrolysate; MMP—mussel meat peptides; GCP—grass carp protein; BSGP—brewer’s spent grain proteins; RPI—rapeseed protein isolate; EWP—egg white protein; OVA—ovalbumin; RDP—rice dreg proteins; BSA—bovine serum albumin; RP—rice proteins; XOS—xylo-oligosaccharides; LSPI—lotus seed protein isolate; +—positive; “-”—negative; “+/-”- positive/negative.

### 2.4. Electrodynamics

Electrohydrodynamic processes like electrospinning or electrospraying are performed by applying an electrical field to a polymer solution that can be spun or sprayed to obtain fibers or particles, respectively [60]. Applying high-voltage electric fields (15–25 kV/cm) extrusion is produced at the end of the needle tip, doubled by the external action of Coulombic forces and internal electrostatic repulsions of the charges accumulated on the surface of polymer solution that induce distortions of the hemispherical droplet into a Taylor cone. When the critical value of the electrical field counteracts the surface tension, fibers or particles are formed [61].

These techniques have found many applications in the food industry where electrospun fibers/particles can be successfully used as protective films or delivery systems [55]. Compared to conventional methods, electrospinning is an excellent alternative for accelerating the formation of Maillard conjugates. The glycation process during electrospinning is affected by the preparation of the polysaccharide–protein fibers/particles and the dry conjugation process known as the annealing process. In the annealing process, the electropun fibers/particles resulted from electrohydrodynamics are maintained under controlled conditions of temperature and relative humidity for a specified time interval [10].

The electrospinning method was found to be very promising for obtaining protein–polysaccharide conjugates with shorter incubation times and higher conjugate yields, as reported by Baier et al. [62]. The authors patented the application of electrospinning as a pretreatment to produce whey proteins–dextran conjugates and reported that the most important parameters that affected fiber formation and conjugation were: dextran concentration, mixture viscosity, protein-to-polysaccharide ratio in the mixture, and time allowed for annealing. The glycation extent increased with increasing annealing time and decreasing the molecular weight of dextran. Good fiber formation and conjugation were reported at 100 kDa dextran, whey proteins:dextran weight ratio above 1:2, annealing time between 4 and 24 h at 60 °C, and a relative humidity of 74%. Kutzli et al. [63] used a combination of electrospinning and dry heating to promote the glycation of the pea proteins–maltodextrins system. Maltodextrins with different dextrose equivalents (2, 10, 21) were tested to investigate their effect on electrospinnability (63.8 kV) and dry glycation (60 °C, 75% relative humidity, 6–24 h). The glycation extent was affected by the maltodextrin composition and incubation time. The addition of two maltodextrins (2 and 10 dextrose equivalent) increased the grafting degree, which reached a maximum after 6 h of heating at 60 °C. The authors did not observe any influence of fiber diameter on the glycation process. More recently, the positive influence of glycation with xylose on the physical properties of gluten/zein nanofibrous films produced by electrospinning was reported by Zhang et al. [64]. The composite gluten/zein electrospun films, further glycated via the Maillard reaction, presented physical properties required for active food packaging applications.

### 2.5. Pressure-based Processing (HP) Techniques

HP technologies are among the most successful nonthermal technologies, well recognized for their advantages of preserving many food products while keeping their freshness. HP treatments represent a good alternative to conventional thermal treatments for modifying the functionality of proteins. Thus, under the action of high pressure, the quaternary, tertiary, and secondary structures of proteins are modified, making them structurally more available to participate in the Maillard reaction and affecting their functionality. Also, pressure favors the formation of the reactive forms of the reducing carbohydrate; however, the addition of carbohydrates with low ionic strength can increase the pressure stability of some proteins, which enables them to rearrange their secondary structure after the pressure treatment [65,66].

Based on the processing conditions applied, Maillard conjugates can be produced with high hydrostatic pressure (HHP) followed by thermal treatment, by high pressure-high temperature (HPHT), also known as pressure-assisted thermal sterilization, by dynamic high-pressure microfluidization (DHPM), or low-pressure homogenization (LPH).

Applied as a single treatment, HHP was shown to be less effective in promoting the formation of Maillard products that enhance the functionality of the proteins. Under the action of pressure, the formation of furfurals or reductones from Amadori rearrangement products is retarded, whereas the breakdown of Amadori rearrangement products is delayed due to nitrogen loss [65]. When HHP is performed at moderate pressure and temperature, the formation of Maillard conjugates is reported. For example, Liu et al. [59] showed that the formation of Maillard conjugates between soybean protein isolate and flaxseed gum was promoted after 3 days of exposure at moderate pressure (100 MPa) and mild temperature (60 °C). The same authors showed that above 200 MPa, pressure had a negative effect on glycation extent. A similar effect was reported in another study, where HHP treatment at 400 MPa was combined for 1 h with mild thermal treatment at 60 °C [67]. It was obtained with a lower effect on the conjugation of β-lactoglobulin with lactose than conventional dry heating (50 °C, 44% relative humidity, 120 h). The authors attributed these results to conformational changes induced by HHP treatment that hindered the accessibility of carbonyl groups of carbohydrate to the free amino groups of the protein.

In comparison with conventional heat treatment, HPHT at 123 °C and 700 MPa for up to 15 min was able to retard the formation of Maillard reaction products in the initial and advanced stages of whey protein–sugar solutions, regardless of the pH investigated [68]. During LPH and DHPM, the protein structure and conformation are affected as a result of cavitation, shearing, turbulence, and heating for short periods, leading to protein unfolding and aggregation [57]. The difference between LPH and DHPM is based on the applied pressure. For high-pressure homogenization, a pump has the ability to deliver at least 100 MPa, regardless of the flow rate [69].

Zhao et al. [57] modified the functionality of pea proteins by using a combination of LPH (80 MPa, 3 passes), US (400 W, 20 min, 70 °C), and wet glycation (20 min, 70 °C) with xylo-oligosaccharides. The authors tested the influence of xylo-oligosaccharides on grafting degree and found that glycation extent increased with increasing xylo-oligosaccharide concentration. However, above a pea proteins:xylo-oligosaccharides weight ratio of 1:3, the grafting degree decreased. Moreover, it was found that the combination of LPH and US was more effective in increasing the glycation degree than the single treatments, due to the LPH’s ability to break down the aggregates and the sonication that induced the partial protein unfolding. On the other hand, glycation was not able to suppress protein aggregation (near isoelectric point) due to the low molecular weight of xylo-oligosaccharides that generated weak steric repulsive forces.

Recently, DHPM was employed as a single treatment for obtaining Maillard conjugates. The exposure of the lotus seed protein–dextran mixture between 40 and 160 MPa accelerated the conjugation, with the highest degree of glycation (17.8%) being reported at 120 MPa [58]. A further increase in pressure had a negative effect on the grafting degree because the molecular forces responsible for maintaining the protein’s spatial structure were broken, leading to protein aggregation. Regardless of the pressure applied in DHPM (40–160 MPa), the degree of grafting was always higher when compared to conventional heating (70 °C, 4 h).

## 3. Functional Properties of Maillard Conjugates

The functional properties of Maillard conjugates are associated with the hydrophilicity or hydrophobicity of proteins that have a significant role in solubility, emulsification, foaming, heat stability, gelation, encapsulation, viscosity, water holding capacity, etc. In this section, the most important functional properties reported for Maillard conjugates prepared with alternative treatments are briefly presented (Table 1).

### 3.1. Solubility

Solubility is one of the most important functional properties of proteins, because in many food applications, proteins should possess good solubility. The solubility is dependent on ion hydration and the electrostatic repulsion between the molecules [49]. As the aggregation and degradation of proteins are associated with their solubility, the lowest solubility is measured under acidic conditions, around the isoelectric pH of the protein. The solubility of proteins can be improved through the Maillard reaction with polysaccharides. Four main arguments for using the Maillard reaction to increase protein solubility were given by Ke and Li [12] as follows: (1) polysaccharide addition changes protein structure by decreasing the sulfhydryl group content, which weakens the protein molecules interaction, which in the end prevents protein aggregation and increases solubility; (2) the hydroxyl groups of polysaccharide addition adsorb on the proteins surface, promoting the interaction of proteins with water while decreasing their surface hydrophobicity; (3) the isoelectric point of the proteins is shifted during Maillard reaction to lower pH values; (4) controlling the degree of Maillard reaction to favor the formation at the protein surfaces of a hydrophobic–hydrophilic dynamic balance.

In most of the studies, the use of alternative treatments was shown to have a positive effect on the solubility of Maillard conjugates. Chen et al. [43] reported that sonication improved the solubility of Maillard peanut protein conjugates at low pH values (3.8) from 45.4% (sonicated protein) to 65.3% for conjugated protein with maltodextrin. Moreover, the authors reported that the solubility at pH 3.8 increased with increasing the grafting degree, with the highest solubility (65.3%) being measured in the sample with a grafting degree of 35%. The use of US for obtaining conjugates based on soy proteins with gum acacia was reported to have better solubility than classical heating and native soy proteins [22]. The solubility of sonicated conjugates (200 W, 80 °C, 40 min) at isoelectric pH was about 35% higher compared to conjugates obtained by conventional heating (80 °C, 24 h). Wang et al. [48] reported that sonication played a positive effect on the solubility of mung bean protein–glucose conjugates, with ultrasonic samples showing a significant increase in solubility at pH values ranging from 4 to 6. The sample exposed to US at 300 W, 20 min, and 80 °C had the highest solubility at most pH values tested in the study (pH 3.0–9.0). Recently, Xue et al. [45] reported that soy protein isolate–cyanidin-3-galactoside conjugate prepared with US had higher solubility and less aggregated structure in aqueous medium. Ma et al. [17] improved, by Maillard conjugation with gum arabic, the water solubility of zein, a protein that is usually dissolved at high alkaline conditions (pH 13.0). The conjugate resulted after US-assisted wet heating (80 °C, 400 W, 25 min) and was well dispersed in the aqueous phase (pH—7.0), transparent without sediment. Increased solubility of whey proteins–gellan gum conjugates prepared by sonication for the all-tested pH range (2.0–10.0) was also reported by Dev et al. [20].

Chang et al. [51] showed that by using MW (440 W, 12 min), the egg white protein–maltodextrin conjugates presented better solubility than egg white protein. Meng et al. [52] tested the solubility of rice dreg protein glycated with sodium alginate using MW treatment (186 W) at pH values between 2.0 and 12.0. The solubility increased under MW treatment, showing the highest solubility at pH > 10, being about 30% more soluble compared to crude protein (pH—12.0). Nasrollahzadeh et al. [34] showed that MW had a positive influence on the solubility of bovine serum albumin–maltodextrin conjugates. On the other hand, the solubility of the conjugates was not affected by the degree of glycation; similar values were reported for conjugates with low (10%), medium (60%), and high (65%) degrees of glycation. The lowest solubility was found at pH 5.0, near the isoelectric point of bovine serum albumin, due to high electrostatic interactions. The effect of MW power, reaction temperature, and time of exposure had a strong effect on protein solubility, as investigated by Cheng et al. [53]. The authors showed that the solubility of the rice protein conjugates increased between 70 and 80 °C, reaching a maximum of about 20% at 85 °C, the equivalent of 150 W. The solubility reached a maximum after 5 min of exposure when using a substrate concentration of 35 mg/mL dextran.

The PEF intensity had a positive effect on the solubility of whey protein–dextran conjugates; the solubility increased from 91.04% to 93.42% with increasing the electric field strength between 15 and 30 kV/cm, shifting the minimum solubility to a more acidic pH value than the isoelectric pH of the protein [38]. The results were attributed to the covalent attachment of highly hydrophilic dextran. Up to 4.5 kV/cm, PEF treatment increased the solubility of bovine serum albumin–starch conjugates to about 86% due to the increased dielectric constant that induced the molecular polarization of the proteins [41].

The positive effect of LPH coupled with US on pea protein–xylo-oligosaccharide conjugate solubility was also found by Zhao et al. [57]. The conjugates had a solubility of around 80–98% in the pH ranges far from the isoelectric point of pea proteins (pH of 3.0, 4.0–10.0), due to the unfolding of protein molecules and increased steric repulsion during US-assisted glycation. The solubility of soy proteins was improved by glycation with flaxseed gum under HHP treatment at 60 °C, reaching a maximum of 86.48% after exposure at 200 MPa, pH 8.0 [59]. At moderate HHP treatment, the quaternary structure of proteins is dissociated, increasing the solubility of the proteins.

In other studies, the antagonistic effect on the solubility of the conjugates when using alternative technologies has been reported. Li et al. [49] investigated the effect of US (500 W, 40 min), conventional wet heating (60 °C, 90 min), and US-assisted glycation (500 W, 60 °C, 40 min) on the solubility of the brewer’s spent grain protein–maltose conjugates. The conjugates prepared by US-assisted glycation had lower solubility (75%) than the wet-heating treatment (80%). The authors explained that the hydrophilic groups of the protein were exposed after US treatment, while the combination with wet heating led to the burial of the hydrophilic group, which increased molecular aggregation while decreasing solubility. Above 4.5 kV/cm, PEF treatment was found to decrease the solubility of bovine serum albumin–starch conjugates (pH 6.0) [41]. The decreased solubility was the result of advanced protein crosslinking, protein aggregation caused by high surface hydrophobicity, and the reaction of advanced glycation end-products with amino groups [9]. MW treatment above 150 W decreased the solubility of rice protein conjugates due to the fast increase in temperature that promoted protein denaturation [53], while Chang et al. [51] found that long exposure at low MW power (250 W, 16 min) or short exposure at high MW power (900 W, 8 min) had an opposite effect on egg white protein conjugate solubility, due to either denaturation or polymerization. HHP treatment above 200 MPa at 60 °C was reported to decrease the solubility of soy proteins glycated with flaxseed gum due to protein denaturation [59].

### 3.2. Emulsification

The protein–polysaccharide conjugates possess excellent emulsification properties that a single protein or carbohydrate does not inherit. However, these properties are strongly influenced by the molecular weight, concentration of the carbohydrate, and ratio between reactants [9]. The hydrophobic residues of conjugates have affinity for the non-polar phase, while the hydrophilic moieties of the carbohydrate interact with the aqueous phase, stabilizing the system by forming a thick steric layer around the biphasic emulsion [20]. Most of the studies conducted on using alternative approaches to obtain Maillard conjugates have shown their positive impact on improving emulsifying properties (Table 1). Their effect on the Maillard reaction is to promote the exposure of both hydrophobic and hydrophilic groups of the protein while creating a more flexible structure that accelerates the migration at the oil–water interface [12].

The glycation of peanut proteins by the US-assisted Maillard reaction was efficient for obtaining conjugates under acidic conditions (pH 3.8), with the samples with a higher degree of glycation presenting better emulsifying properties due to lower surface hydrophobicity [43]. Zhang et al. [46] reported that the combined US treatment and wet heating Maillard reaction (450 W, 90 °C, 30 min) improved the functionality of β-conglycinin–maltodextrin conjugates in terms of emulsifying activity and stability, due to the increased degree of grafting (32.73%) of the conjugates. The glycation of mung bean protein–glucose conjugates treated by US and wet heating (300 W, 10 min, 80 °C) was found to speed up the conjugation process while improving the emulsifying properties [48]. The positive impact of sonication on the emulsification properties was reported for other conjugates, such as whey proteins–gellan gum conjugates [20], while Ma et al. [17] highlighted that zein–gum arabic conjugates obtained by combining sonication with wet heating (400 W, 25 min, 80 °C) were effective emulsifiers. Recently, Ren et al. [31] reported that US treatment (300 W, 80 °C, 35 min) was able to produce Maillard conjugates between fish protein hydrolysate and xylose with a water content of 10%. The emulsifying properties of the Maillard conjugates assisted by US treatment were higher by about 50% compared to the control sample.

The use of MW for producing Maillard conjugates with increased emulsifying properties was reported as well. For example, Hu et al. [33] showed that the emulsifying activity and emulsion stability of ovalbumin glycated with glucose increased with increasing MW power and processing time. After 15 min of glycation at 640 W, the emulsifying activity and emulsion stability were about 340 m^2^/g and 90 min, respectively. The authors concluded that MW was able to produce conjugates with better emulsifying properties than conventional dry heating. The emulsifying stability of rice proteins–sodium alginate conjugates prepared under MW heating decreased in the pH interval of 2.0 to 6.0 and increased between 6.0 and 12.0, as recently reported by Meng et al. [52]. The conjugates obtained with MW treatment presented higher emulsifying stability (75.43%, pH—12.0) than native protein. In addition, the authors mentioned that the change in emulsifying stability under different pH conditions indicates an improved emulsifying capacity and a positive correlation between the emulsifying stability and solubility of Maillard conjugates. Improved emulsifying activity and stability under MW treatment were reported for other glycated proteins like bovine serum albumin–maltodextrin conjugates [34], rice protein–dextran [53], etc. The positive effect of PEF on the emulsifying properties of whey proteins–dextran conjugates was reported by Sun et al. [38]. The emulsifying properties increased with increasing the pulsed electric field strength (15–30 kV/cm), with the highest emulsifying activity and stability being 55 m^2^/g and 19 min, respectively. The authors attributed the enhanced emulsifying properties of the glycoprotein-stabilized emulsions to the hydrophobic protein moiety that is firmly attached to the oil droplet surface and the hydrophilic character of covalently linked polysaccharides that act as a steric stabilizing layer for preventing the coalescence of the oil droplets. The use of electrospinning (63.8 kV) as a pretreatment for obtaining pea protein–maltodextrin conjugate fibers with improved functional properties was investigated recently by Kutzli et al. [55]. Electrospinning followed by dry glycation (70 °C, 75% relative humidity, 24 h) increased the emulsifying activity and stability of the conjugates, which were less susceptible to pH changes.

Conjugates with superior emulsifying properties were obtained with DHPM. Zheng et al. [58] tested the contribution of pressure between 40 MPa and 160 MPa to obtaining Maillard lotus seed protein–dextran conjugates. Highly stable emulsions were obtained out of lotus seed protein conjugates prepared at 120 MPa due to the strong shear force and pressure induced during DHPM that led to faster adsorption of the protein to the oil/water interface. On the other hand, above 160 MPa, the emulsifying activity and stability of the conjugates decreased due to stronger mechanical and thermal effects induced during DHPM that caused the denaturation and aggregation of the proteins and reduced their contact with oil.

When combining more than two treatments, an additive effect on emulsification activity was reported. For instance, Zhao et al. [57] used LPH and US followed by wet heating to obtain pea proteins–xylo-oligosaccharide conjugates with superior emulsifying properties. When compared to native protein, the combined treatment followed by conventional heating improved the emulsifying activity of pea protein isolate by about 32%. On the other hand, the effect of LPH and US on the emulsifying stability of conjugates was similar to that of the native protein. The authors correlated the low emulsifying activity of the conjugates with the low number of monosaccharides in the xylo-oligosaccharides, which decreased the steric repulsion.

### 3.3. Foaming

Foaming characteristics in terms of foaming capacity and stability play an important role in determining the quality of various food products like ice-cream, cakes, toppings, and mayonnaise due to their contribution to structure formation [5]. Many reports suggest that conjugates obtained by the Maillard reaction with alternative treatments possess superior foaming properties compared to native proteins. However, as in the case of other functional properties, the foaming properties depend on the molecular weight and structure of reactants, reactant ratio, and reaction time, which affect the glycation extent.

The whey proteins–gellan gum conjugate prepared by sonication showed enhanced foaming capacity compared to whey protein isolate alone [20]. Sonication (500 W, 40 min, 10 °C) combined with wet heating (90 min, 60 °C) promoted the formation of spent brewer’s grain protein—maltose conjugates with improved foaming capacity and stability, due to enhanced hydrogen bonding ability and hydrophobic interaction between protein molecules [49]. The effect of US (300 W, 80 °C, 35 min) on obtaining protein hydrolysate–xylose conjugates in medium with 10% water content was investigated by Ren et al. [31]. Foaming activity and stability of the conjugates were higher by about 6% and 22% compared to sonicated protein hydrolysate and were attributed to the increased viscosity of glycated protein, which enlarged the polymer layer and delayed water loss.

On the other hand, the sonication of bovine serum albumin at 300 W, followed by wet glycation with dextran at 90 °C for 150 min, was found to have an antagonistic effect on protein foaming stability compared to the protein sample exposed to US, as recently reported by Cui et al. [21]. The decreased foaming stability was attributed to decreased surface hydrophobicity after the glycation reaction, which reduced the affinity to the air–water interface. However, when compared to conventional heating, US played a positive influence on both the foaming capacity and stability of bovine serum albumin conjugates.

The foaming capacity and stability can be affected by the structural flexibility of the proteins. Proteins with flexible structures, like casein, exhibit excellent foaming properties as they rearrange rapidly at the air–water interface [21]. The mechanical effect of ultrasonic cavitation increases the foaming properties of the casein conjugates even more. This phenomenon is associated with the exposure of non-polar groups in the aqueous phase of casein, which in turn improves its surface activity.

Chang et al. [51] showed that the MW treatment up to 600 W of the egg white protein-maltodextrin conjugates, compared to the control group, improved the foaming ability and stability by about 36.7% and 8.2%, respectively. The authors explained that the MW treatment promoted the exposure of hydrophobic patches and hydroxyl groups, which further favored the hydrophobic and hydrogen bonding interactions between protein molecules. Nasrollahzadeh et al. [34] reported that conjugates with a high degree of glycation obtained under MW heating presented superior foaming capacity compared to the samples prepared with conventional heating. The authors explained that conjugates with a high degree of grafting have a more flexible structure, helping the protein to rapidly rearrange at the air–water interface and create foam. The effect of LPH combined with US on the foaming properties of pea protein–xylo-oligosaccharide conjugates was reported by Zhao et al. [57]. First, pea protein solution was treated by LPH at 80 MPa for 3 times, then mixed with xylo-oligosaccharide at a weight ratio of 1:1, and then exposed to US at 400 W for 20 min at 70 °C. The use of LPH as a pretreatment followed by glycation under US exposure resulted in a synergistic effect regarding the improvement of the foaming properties of conjugates. Thus, the foaming capacity increased from 30.78% to 69.33%, whereas the foaming stability increased from 23.44% to 48.78%.

### 3.4. Thermal Stability

Enhanced thermal stability of conjugated proteins can be obtained by using anionic polysaccharides through steric and electrostatic repulsions. The polysaccharide moiety, via the crowding effect, can reduce the solvent availability of protein, forcing it to adopt a compact and steady-state structure that prevents protein aggregation [70]. Moreover, performing the Maillard reaction at high temperatures can damage the protein structure, with negative consequences for both the functionality and bioactivity of the final products. The effect of using alternative treatments on improving the heat stability of the protein conjugates was reported in several studies. For example, Dev et al. [20] evaluated the thermal stability of whey proteins–gellan gum conjugates obtained by US exposure. Compared to conventional wet heating, sonication (300 W, 50 °C, 60 min) resulted in a higher grafting degree that increased the heat stability of the resulting conjugates by a factor of 2.96. On the other hand, when increasing the temperature from 50 to 90 °C, conjugates obtained by conventional heating had a higher exposure to the unfolded protein and displayed a low glycation degree, producing protein aggregation and precipitation. The results are in line with those reported by O’Mahony et al. [13], who indicated that the heat stability of conjugated whey proteins is dependent on the grafting degree, carbohydrate chain length, and glycation sites of the protein, with polysaccharides providing better protein heat stability. The aqueous dispersions and solid powders of soy protein isolate–cyanidin-3-galactoside conjugates obtained after sonication (106 W/cm^2^, 20 min) presented better thermal stability than that of soy protein isolate [45]. The heat stability of zein was improved by the gum arabic addition during the US-assisted Maillard reaction (400 W, 25 min, 80 °C), due to the covalent attachment of gum arabic to zein [17]. Nasrollahzadeh et al. [34] demonstrated that MW has a positive effect on the thermal stability of bovine serum albumin–maltodextrin conjugates. On the other hand, stability was affected by the grafting degree; conjugates with a lower degree of glycation presented higher thermal stability. However, regardless of glycation degree, conjugates obtained with MW treatment presented better thermal stability than conjugates obtained with conventional heating.

The PEF treatment inhibited the aggregation of whey protein–dextran conjugates (at pH 4.0, 5.0, 6.0, and 8.0), as detailed by Sun et al. [38]. The thermal stability was assessed at 80 °C for 20 min on the conjugates resulting from PEF treatment. The aggregation inhibition was attributed to reduced hydrophobicity during PEF glycation and steric hindrance due to the covalent attachment of dextran, which suppressed intermolecular association among proteins.

The use of LPH, US, and LPH-US increased the thermal stability of pea protein–xylo-oligosaccharide conjugates [57]. In particular, the LPH-US combined treatment was the most efficient in improving the thermal stability of the conjugates due to the higher grafting degree. The thermal denaturation of LPH-US conjugates increased by about 20% compared to pea proteins. It seems that at high temperatures, the presence of xylo-oligosaccharide moieties on the surface of pea proteins inhibits their unfolding due to steric hindrance effects.

### 3.5. Gelation

There are limited studies reported in the literature that investigate the influence of alternative treatments on the gelation properties of Maillard conjugates. By adding polysaccharides during the Maillard reaction, the protein structure is modified due to increased hydrophobic interactions between protein molecules. During gel formation, these interactions promote protein aggregation and cross-linking, while the covalent bond formed during the Maillard reaction strengthens the gel structure and prevents fragmentation [12].

Zhao et al. [25] applied a combination of US and wet heating to obtain conjugates from soy protein isolate and maltodextrin. Exposure for 5 min to the US was recommended by the authors for producing conjugate acid-induced gels with superior gel strength and water holding capacity than the control group. On the other hand, prolonged ultrasonic treatment (15 or 25 min) weakened the gelation properties. More studies are needed in order to understand the effect of the alternative treatments on the gelation properties through the Maillard reaction.

### 3.6. Encapsulation

The efficiency of protein conjugates prepared by conventional heating for encapsulating hydrophobic and hydrophilic compounds has been reported in many studies; a comprehensive review on this topic was recently reported by Nooshkam and Varidi [70]. Despite the huge potential of involving alternative treatments in preparing protein conjugates as protective materials for sensitive compounds, there are limited studies reported on this topic. The application of US and glycation to improve the functional properties and encapsulation efficiency of anthocyanins in proteins was investigated in a recent study conducted by Cui et al. [21]. The bovine serum albumin or casein solutions were first exposed to US (300 W) and further glycated with dextran at a protein:carbohydrate ratio of 1:2.5 (*w*/*w*). The US parameters tested were amplitude (0–80%), time (0–15 min), and pH (7.0–11.0), whereas wet heating was performed at 90 °C for 150 min in a water bath. Ultrasonic pretreatment increased the grafting degree of the conjugates, promoting protein glycation. On the other hand, the grafting degree of bovine serum albumin was higher than of casein, due to bovine serum albumin helical structure that during US exposes more glycation sites. The US pretreatment decreased the pH, an indication that amino acids are consumed, leading to the formation of acids like formic acid, acetic acid, glyoxal, etc. For testing their potential for anthocyanin encapsulation, the conjugates were further mixed with a carboxymethyl cellulose solution containing the anthocyanins. The resultant mixture was exposed once again to US at 300 W to promote complex assembly, and then the complex was evaluated for thermal stability and antioxidant activity. Although the authors confirmed the formation of a relatively uniform and stable system in which anthocyanins are embedded, the encapsulation efficiency and retention were not measured. The thermal stability of the complex was higher than the control, whereas the antioxidant activity of the anthocyanins was not affected when loaded into the complex. An interesting phenomenon was that after heat treatment of complexes containing anthocyanins, the antioxidant activity was improved compared with the anthocyanin sample, indicating the protective effect of the complex containing anthocyanins during thermal treatment.

## 4. Biological Properties of Maillard Conjugates

In the last decades, the application of alternative technologies for obtaining Maillard conjugates has been on the rise due to their remarkable potential to improve their bioactivity in terms of antioxidant, antimicrobial, antihypertensive, anti-inflammatory, antimutagenic, and bifidogenic properties. Frequently, these alternative treatments accelerate the Maillard reaction; therefore, the formation of compounds with improved bioactivity is achieved in a shorter time than in conventional wet or dry heating. Among the biological properties, antioxidant activity is one of the most investigated properties of Maillard conjugates prepared with alternative technologies. Although the effect of conventional heating on other biological activities was tested, as recently reviewed by Nooshkam et al. [4], the potential of alternative technologies was not yet demonstrated and requires more research efforts.

### Antioxidant Activity

The antioxidant mechanisms of the Maillard reaction are attributed mainly to free radical scavenging, scavenging reactive oxygen species, and metal ion chelation [12]. The reductone intermediates formed in the initial stage of the Maillard reaction break the free radical chain reaction by providing hydrogen atoms. Moreover, these reductones have the potential to prevent the formation of peroxides due to their capacity to react with specific peroxide precursors. Melanoidins formed in the advanced stages of the Maillard reaction have anionic properties that promote the chelation of metal ions while limiting oxidation reactions. The alkaline environment promotes the formation of melanoidins to a greater extent than neutral and acidic conditions [12].

In order to maximize the potential of combining alternative with conventional technologies for improving the biological properties of the Maillard conjugates, it is essential to perform the glycation process under controlled conditions because, in the advanced stages of the Maillard reaction, the bioactivity is negatively affected. Also, the controlled conditions will prevent the formation of toxic compounds (e.g., heterocyclic amines, acrylamide, etc.) that induce potential safety issues [71]. The antioxidant properties of Maillard conjugates are dependent on the amino acid composition, sequence, and chain length of peptides, as well as the stability of the peptide bond against heat treatment, time, and temperature [72]. Recent studies indicated that the formation of melanoidins during US exposure in the temperature range of 55–75 °C was always higher than conventional heating, indicating the promising potential of sonication in promoting the interaction between dicarbonyl products and L-serine in the formation of melanoidins, which will increase the antioxidant activity of the protein conjugates [73].

The antioxidant properties of whey protein–gellan gum conjugates prepared by sonication (300 W, duty cycle 50%, 60 min) were reported by Dev et al. [20]. The authors measured the ABTS and DPPH radical scavenging activity and reducing power capacity of the US-assisted Maillard products and showed that samples prepared by US treatment had a higher antioxidant activity compared to the native samples. These results were attributed to the exposure of more hydrophobic amino acids such as proline, alanine, valine, leucine, isoleucine, and phenyl-alanine that stabilized the free radical by donating the proton to electronically deficient unstable radicals. The antioxidant activity of zein-gum arabic Maillard conjugates was improved by using sonication and heating (400 W, 25 min, 80 °C), due to the sonication ability to accelerate the Maillard reaction, generating antioxidant reaction products [17]. Abdelhedi et al. [26] reported that US had a strong impact on enhancing the antioxidant activity of Maillard conjugates obtained between low-molecular-weight peptides and sucrose. The antioxidant activity of the conjugates prepared with US treatment was twofold higher compared with the control sample. Similar results were reported for protein hydrolysates from mussel meat glycated with glucose/xylose under sonication treatment [30], for grass carp peptides grafted with glucose [28], and for whey protein peptide–galactose conjugates [27]. The positive impact of MW on the antioxidant potential of the ovalbumin–glucose mixture was highlighted by Hu et al. [33]. The antioxidant activity measured in terms of reducing power, DPPH radical scavenging activity, and TEAC of the glycated samples increased with increasing reaction time, heating temperature, and microwave power. The results indicated that solid-state glycation under MW exposure (640 W, 15 min) was more efficient to improve protein antioxidant activity compared to conventional dry heating. The antioxidant activity of bovine serum albumin–maltodextrin conjugates prepared under MW with different levels of glycation was explored by Nasrollahzadeh et al. [34]. The highest antioxidant activity (39.74%) was measured in samples with a high degree of glycation, due to the formation of melanoidins that can act as potential antioxidants. Compared to conjugates obtained by conventional heating, MW heating was able to increase the antioxidant activity of conjugates with a high degree of glycation by about 28.5%.

The positive effect of PEF on the antioxidant activity of Maillard conjugates was reported in some studies. For example, Guan et al. [39] tested the antioxidant activity of bovine serum albumin–dextran conjugates obtained by using an electric field intensity of 10 kV/cm and 20 kV/cm, respectively. When compared to the control sample (protein exposed to PEF), the antioxidant activity of the Maillard conjugates increased with increasing electric field intensity, reaching a maximum radical scavenging activity of about 17%. The results were attributed to the molecular weight and conformation of cross-linked proteins that, in the presence of dextran, form Maillard products with hydrogen-donating properties. On the other hand, Wang et al. [54] combined PEF at a higher electric field intensity (40 kV/cm) with wet glycation for the glycine–glucose system and reported that PEF pretreatment enhanced the Maillard reaction, contributing to the formation of conjugates that increased the antioxidant activity to 39.36%.

Recently, Kwak et al. [56] combined electrospinning with dry glycation (100 °C, 4 h) to obtain gelatin nanofibers containing glucose, sucrose, and fructose. Glycation extent and antioxidant activity were affected by the type of sugar used in Maillard conjugation; the highest grafting degree and antioxidant activity were measured in gelatin–fructose nanofiber conjugates. Moreover, the addition of sugar increased the stability of nanofibers against hydrolytic degradation and enhanced the physico–chemical properties of the gelatin nanofibers. The strong positive contribution of LPH, US, or LPH-US to obtaining pea protein–xylo-oligosaccharide conjugates with superior antioxidant activity was reported by Zhao et al. [57]. Moreover, the conjugates with improved antioxidant activity were more effective at inhibiting lipid oxidation than native proteins due to the formation of reductones. The authors recommended combining LPH and US treatments as an effective way to prepare conjugates that can be used as antioxidants in food.

## 5. Limitations and Perspectives

Despite the fact that alternative technologies like ultrasound, microwave, pulsed electric fields, electrospinning, and high pressure facilitate the formation of Maillard products that improve the functionality of proteins, in many aspects, their use is still challenging. In order to maximize their contribution to the formation of Maillard reaction compounds with superior functional and biological properties, the proteins and carbohydrates, concentration, and ratio should be selected according to the specific needs. For example, some protein conjugates have better foaming or emulsifying properties that recommend them for confectionaries, while others show stronger antioxidant activity and are suitable as encapsulating agents. On the other hand, a higher concentration of protein could promote protein degradation, with negative consequences for most functional properties. The selection of the alternative technology for assisting the Maillard reaction is also dependent on the desired functionality of the proteins. For example, electrospinning is well known for its potential to obtain conjugates to be used as films with high antioxidant activity for food packaging applications. Although conventional wet and dry heating continue to be the most applied strategies for tuning protein functionality through the Maillard reaction, the number of applications that use alternative technologies, and in particular ultrasound treatment, is on the rise. Although the structure–function mechanism of the Maillard conjugates resulting from the alternative technologies is not yet completely elucidated, most of them highlight positive contributions to improving protein functionality.

Moreover, many alternative technologies have shown good results in improving the functional and biological properties of Maillard conjugates when used as pretreatments or in combination with conventional heating, while others like microwave heating, ultrasound, dynamic high-pressure homogenization, or pulsed electric fields were found to have good potential to be used as alternatives to conventional heating. On the other hand, the impact of these technologies has to be carefully investigated to prevent either the degradation of proteins or the formation of toxic compounds. More research efforts should be engaged to determine the impact of these alternative technologies on the safety and nutritional profile of the conjugates.

Although many alternative technologies employed for assisting the Maillard reaction have shown a positive influence on the functionality of the proteins, in most cases, the study of their contribution to the biological properties was limited to antioxidant activity. Thus, additional studies are needed to elucidate their impact on other biological properties like mutagenicity, antimicrobial activity, digestibility, etc. It was shown that the controlled hydrolysis of proteins prior to conventional glycation has a positive effect on the functionality of the resulting conjugates. However, knowledge on the effect of alternative technologies on the functionality and bioactivity of these peptide-based conjugates is limited and requires future research studies.

From many studies showing applications of combined treatments to produce Maillard conjugates, it was not possible to accurately identify if the effect of alternative treatments on functionality was additive or synergistic, mostly due to incomplete or inappropriate design studies. Therefore, it has to be further investigated in comparison with untreated native protein and with a mixture of native protein and carbohydrate before conjugation to identify what type of positive effect it has on the functionality and bioactivity of the Maillard conjugates.

The last but not least, the use of ultrasound, microwave, pulsed electric fields, or high pressure as pretreatment or in combination with conventional heating could promote the formation of Maillard reaction compounds with desired functionality and bioactivity in a shorter time and with reduced costs and a lower environmental impact than employing only conventional heating. Although the use of some alternative technologies like pulsed electric fields and microwaves as single treatments to obtain Maillard conjugates with superior functionality is very promising, their use is still limited due to high initial capital costs, whereas the feasibility at the industrial scale has to be demonstrated.

## 6. Conclusions

This review presents the current status of using alternative technologies based on ultrasound, microwave, pulsed electric fields, electrospinning, and high pressure for improving protein functionality and bioactivity through the Maillard reaction in terms of solubility, emulsifying, foaming, heat stability, gelation, encapsulation, and antioxidant activity. The main benefit of using these alternative treatments relies on their ability to speed up the Maillard reaction rate while minimizing the formation of undesired compounds. This review showed to what extent the use of alternative treatments could replace, or at least complement, the traditional conventional glycation methods for tuning the functional and biological properties of the proteins.
